# Evaluating the accuracy of intraoral scanners across varied depths and widths preparation for CAD/CAM post and core

**DOI:** 10.3389/fdmed.2026.1691514

**Published:** 2026-04-29

**Authors:** Dinesh Rokaya, Ahmad Al Jaghsi, Mazen Abdulmounem Masri, Nada Mohammed Hussein, Ghazal Tamer Said, Tala Akhras

**Affiliations:** 1 Clinical Sciences Department, College of Dentistry, Ajman University, Ajman, United Arab Emirates; 2 Center of Medical and Bio-Allied Health Sciences Research, Ajman University, Ajman, United Arab Emirates; 3 Department of Prosthodontics, Gerodontology and Dental Materials, Greifswald University Medicine, Greifswald, Germany

**Keywords:** accuracy, CAD/CAM, digital dentistry, digital scan, intraoral scanner, post and core

## Abstract

**Objectives:**

This study aimed to investigate the accuracy of two intraoral scanners at different post space preparation depths and widths for CAD/CAM fabricated post and core fabrication.

**Methods:**

A total of 36 extracted anterior teeth were used in this study, which were divided into 6 groups according to the different widths and depths of post space preparations. Two operators carried out the intraoral scanning using two scanners: Planmeca Emerald and 3Shape TRIOS 4. Furthermore, the inEos X5 extraoral laboratory scanner was used as a reference. 3D point cloud processing software was used to analyze the impact of different widths and depths on the scanners' accuracy.

**Results:**

After ensuring examiner blinding, it showed that there was no significant difference between the 3Shape and Planmeca scanners. Additionally, longer posts resulted in lower scanning accuracy across the board, whereas wider posts tended to produce results that were more accurate.

**Conclusions:**

This study found that there was no difference in accuracy between the 3Shape and Planmeca scanners. The distribution of scanning accuracy at different post space preparation depths and widths showed that longer posts resulted in lower scanning accuracy, whereas wider posts tended to produce results that were more accurate, which provides information for the CAD/CAM post and core fabrication.

## 1 Introduction

In recent years, digital dentistry has transformed traditional dental practices, presenting unmatched precision, efficiency, and patient comfort (1). Improved therapeutic interventions, treatment planning, and diagnostics have become possible as a result of workflows in dentistry that incorporate state-of-the-art technologies (2). Together with these developments, intraoral scanners (IOS) have become essential instruments that enable dentists to get precise, 3D images of the oral cavity (3, 4). IOS has improved treatment outcomes and patient satisfaction by replacing traditional impression-taking techniques and modernizing the production of various dental prostheses, such as crowns, bridges, and implants (5, 6). In addition, digital dentistry has gone further with the use of computer-aided design (CAD) and computer-aided manufacturing (CAM) technologies, making it easier to fabricate custom restorations with a high degree of accuracy and precision (1). The accuracy of a scanner is the ability of a measurement to match the actual value, and the precision of a scanner is the ability of a measurement to be consistently reproduced (7). The accuracy can also be termed as trueness. The IOS presents some distortion and most often presents stitching errors (8, 9). The distortion of the scan varies with the scanners, techniques, and pattern of scan. The full-arch intraoral scans can be obtained by various scan strategies, with the segmental scan and merge methods. The segmental scan and merge methods using two scan parts can be a reliable alternative to the single scan method for full-arch intraoral scans (10). But when more than three segmental scans negatively affect the accuracy of the complete arch scan.

Following the endodontic treatment, an appropriate restoration of the tooth establishes a coronal seal and protects the remaining dental structure. For the teeth with significant damage, posts and cores serve to stabilize and retain the crowns (11). Several digital techniques have been used in the making of the post and core, which not only reduce patient chair time but also eliminate the necessity for temporary restorations and repetitive adjustments, thus improving treatment efficiency and minimizing overall treatment duration (1, 12, 13). However, as with any technological evolution, the efficacy, accuracy, and precision of IOS require thorough evaluation, particularly in significant applications such as CAD/CAM post and core fabrication (14–16).

Digital fabrication for the post can be done by various techniques: direct digital technique by directly scanning the post, semi-direct technique by scanning the root canal impression, semi-direct technique by scanning the resin pattern, and indirect fabrication of the post. Direct digital technique involves directly scanning the post space using an IOS after root canal treatment, and designing using the CAD software (17). The semi-direct technique involves scanning the root canal by injecting polyvinyl siloxane into the prepared root canal and then making an impression with heavy-body impression material (18, 19). In the semi-direct technique by scanning the resin pattern, the auto-polymerizing acrylic resin (GC Pattern Resin) post (20). Following the scanning and computer designing, the post-and-core can be milled using different materials, such as fiber-reinforced composite (21) and fiberglass (22). This is the most practical method of fabrication of the post.

The evaluation of marginal and internal adaptation in dental restorations is crucial for the long-term success and functionality. Different techniques in measuring the internal gap in post and core include the silicone replica technique, micro-computed tomography [micro-CT], digital technique, semi-digital technique, optical coherence tomography (OCT), and radiograph technique (23–28). Among various methods, the silicone replica technique stands out as a reliable and commonly utilized approach. Similarly, the digital technique involves the fabrication of posts and cores using the digital workflow. The ruler tool in Adobe Photoshop can be used to analyze the images (26). In a semi-digital technique of fabricating customized posts and cores, digital data using light scanning is used to acquire data from a manually created wax pattern manufactured using the traditional lost-wax procedure.

With this amount of diversity and complexity, thorough assessments of intraoral scanner accuracy become vital, particularly in the setting of CAD/CAM post and core preparations. This study aimed to investigate the accuracy of two intraoral scanners through varied depths and widths of tooth preparations, offering valuable insights into their clinical performance and potential limitations.

## 2 Materials and methods

### 2.1 Study samples and study groups

The study protocol was approved by the Institutional Review Board of the Ajman University (protocol code D-H-S-2023-NOV-20-14 and approval date 7 December 2023). From a total of 150 extracted anterior teeth, 36 intact teeth were selected according to the selection criterion: sound teeth, intact root, straight canals, single canal, and fully formed apex. The teeth were examined radiographically to evaluate the canals, to assess the anatomy, and to identify the canal curvature. Additionally, the teeth were examined using a stereomicroscope under high magnification to evaluate each tooth according to the criteria and check for the presence of any fractures or cracks. Teeth were excluded due to curved canals, incomplete apex formation, or the presence of cracks/fractures observed under the stereomicroscope. Hand scaler was used for the root surface debridement of each sample, followed by disinfection with 5% NaOCl solution for 24 h at 4 °C, and afterward, they were stored in 0.1 thymol solution (29, 30).

The study groups consisted of 9 groups of different widths and depths of post space preparation. The three widths used were 1.8, 2.1, and 2.3 mm, and the lengths were 6, 8, and 10 mm. To classify the teeth, each tooth was evaluated radiographically using the digital ruler to measure the length and width of the canal; accordingly, the samples (teeth) were prepared.

### 2.2 Sample preparation

After classifying the teeth according to the radiograph, the anatomical crowns of the teeth were sectioned 2 mm coronal to the cementoenamel junction (CEJ), and only the roots were used for the study. Each tooth was sectioned to the specific length of the group it belonged to, and this step was done by using a micromotor with a straight handpiece and diamond-coated metal (double-sided) cutting discs. The fine diamond discs permit precise cuts without affecting the tooth's integrity or producing any irregularities. The Periodontal Williams probe was used to clinically check the canal orifice after discing the teeth.

### 2.3 Post-Space preparation

**Figure 1:**
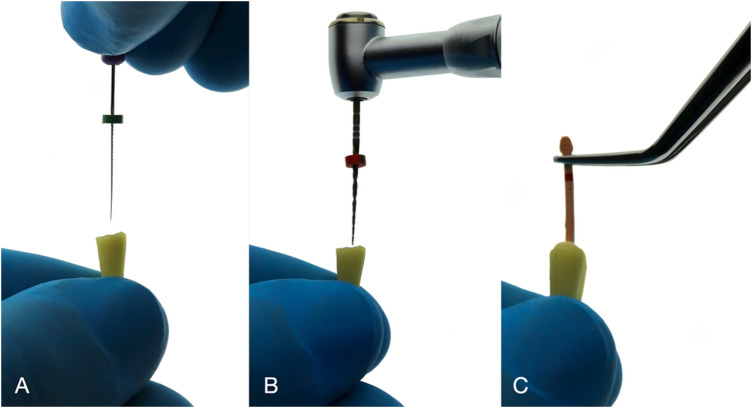
Steps in endodontic treatment. **(A)** Working-length determination using K-File size 10. **(B)** Canal Instrumentation using WaveOne Gold single-file reciprocating system (primary). **(C)** Obturation using the single cone technique (WaveOne Gold Gutta Percha Points).

Following sample preparation, cleaning, and shaping of the canals was done. Canals were irrigated with 1.75% of sodium hypochlorite (NaOCl) and saline using endodontic irrigation needles (Endo-Top, Cerkamed, Stalowa Wola, Poland). Patency of the canals was checked with K-Files size 10. After establishing the working length with the initial apical file for each tooth (Figure 1A), the canal system was shaped and prepared using the WaveOne Gold single-file reciprocating system (WO; Dentsply Maillefer, Ballaigues, Switzerland) (Figure 1B). To standardize all canal systems, all teeth were instrumented with the same large-size file. We used an endodontic instrument of size 25 (ISO 025) K-file and a rotary file**,** which has a tip diameter of 0.25 mm.

After root canal instrumentation, all the canals were dried with absorbent paper points (Meta Biomed Co, Ltd, Chungbuk, Korea), then obturated with a single cone obturation technique using WaveOne Gold Gutta Percha Points (DentsplyMaillefer, Ballaigues, Switzerland) that match the size of the prepared canal (Figure 1C). In all the samples, AH Plus (Dentsply De Trey, Konstanz, Germany) was used as an endodontic sealer. After endodontic preparation and obturation, the gutta-percha cone was cut by using the Endodontic Gutta-percha Cutter (Elements™ IC Backfill Unit, Kerr, California, USA). Teeth were kept at 37 °C and 100% humidity for 14 days in order to give the sealer time to fully set (31).

To achieve the desired widths and lengths of the teeth, specific burs were used. Tapered diamond burs with widths of 1.8, 2.1, 2.3 mm and lengths of 6, 8, 10 mm were used (Komet, Germany). All burs had a taper of 4 degrees.

**Figure 2:**
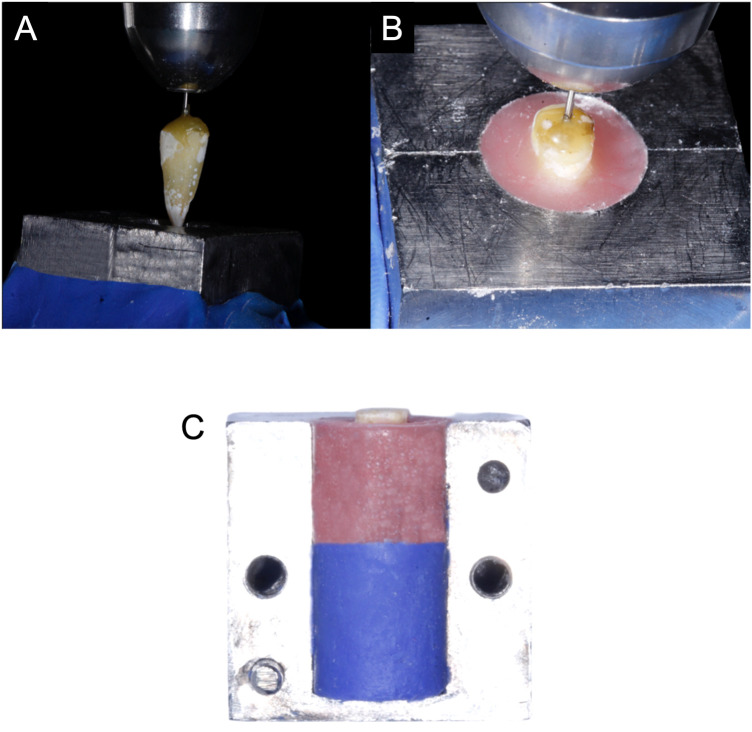
Preparation of the post space. **(A)** Tooth positioned in the center using sticky wax and Gates Glidden. **(B)** The tooth positioned with its surface raised 2–3 mm resembling the ferrule effect. **(C)** PVS Putty (Type 0 ISO) filled halfway to reduce material wastage.

Hydrorise putty (Zhermack SpA, Badia Polesine, Italy) was mixed and adapted onto the modeling table MT3 of the milling machine (AF 350, Amann Girrbach, Koblach, Austria) with a metal mold placed in the center. The metal mold was used to achieve a standardized shape and size for the acrylic blocks. To minimize material wastage, a small amount of hydrorise putty was added to the metal mold, filling it halfway (Figure 2C).

Before commencing the acrylic block fabrication process, the tooth was positioned according to its long axis (Figure 2A). Size 2 (0.7 mm) Gates Glidden drills (Mani Inc. Japan) were inserted into the gutta-percha partially (not to the full targeted preparation depth) to ensure clear long-axis orientation. Sticky wax was heated and applied between the drill and tooth to create a tight seal, preventing movement or disengagement. The assembled Gates Glidden drill and tooth were then placed into the bur slot of the milling machine.

Before mixing the acrylic, a thin layer of separating medium was applied to the metal mold to facilitate easy removal of the acrylic after setting. The tooth was positioned in the metal mold with its surface raised 2–3 mm above the metal's surface, resembling the ferrule (Figure 2B). This adjustment was achieved by manipulating the “pivoted lever for drilling” and turning the “fixation screw for height adjustment,” followed by locking the desired height with the “locking screw for jointed arm” of the milling machine.

Subsequently, an appropriate amount of Orthoplast Liquid (Vertex-Dental B.V., Soesterberg, Netherlands) was mixed with Vertex Orthoplast Powder to achieve a thin consistency. This mixture was poured into the mold, and any excess was carefully removed. The tooth was positioned, and the acrylic block was left to set (32).

For accurate and precise post space preparation, the procedure was carried out using the precision milling machine to eliminate potential human errors, such as hand tilting, which could result in deviation from the long axis of the tooth and potential perforation.

The post-space preparation was performed using a standardized drill (Taper 4, angle of convergence 8°). A size 2 (0.7 mm) Gates Glidden drill was used to remove the gutta-percha of the obturated tooth, leaving 5 mm of apical gutta-percha. The micrometer of the milling machine was adjusted to achieve the desired depth of penetration. Gutta-percha was removed from the 2/3 length of the root, keeping a minimum of 5 mm at the apex. The final post-space exhibited a coronal diameter of 2.3 mm at the orifice and extended to a depth of 10 mm along the canal's long axis. Owing to the 4° taper per side, the apical diameter was approximately 0.9 mm.

Subsequently, peeso reamers (Azdent, Henan, China) were sequentially employed, up to the maximum size that was smaller apically than the apical diameter of the final bur, such that we ensured that the outline of the preparation was created by the final bur. This was determined by the formula: Diameter—tan (4) × Length, where the diameter is the diameter of the bur to be used, 4 represents the degree taper of the bur, and length represents the length to which the tooth is to be prepared. For instance, for a tooth with a diameter of 2.3 mm and a length of 10 mm, the maximum allowed peeso reamer size was calculated to be 1.6 mm. Consequently, only reamer sizes up to 1.5 mm were used. The micrometer was adjusted according to the post depth to maintain the desired apical gutta-percha length.

Saline irrigation was continuously directed onto the reamer to prevent overheating and ensure adequate lubrication. To finalize post space preparation with the final bur, a designated bur (with the aimed width and length) was selected and placed into the milling machine's bur slot. The bur tip was positioned precisely above the orifice center. After adjusting the micrometer to the required depth, the magnet of the modeling table was locked, and saline irrigation was applied. The bur was allowed to enter with light pressure and copious irrigation. At halfway, the bur was cleaned to remove debris that could hinder reaching the full length. The tooth was irrigated with saline midway to prevent overheating.

Multiple entries with the bur were avoided to prevent unnecessary widening of the post space. After preparation, an x-ray was taken to confirm proper alignment along the tooth's long axis. Following x-ray confirmation, three equal grooves were created on the tooth using a carbide straight fissure bur, each measuring 0.5 × 0.5 mm, to facilitate later superimposition in the 3D software.

### 2.4 Intraoral scanning

**Figure 3:**
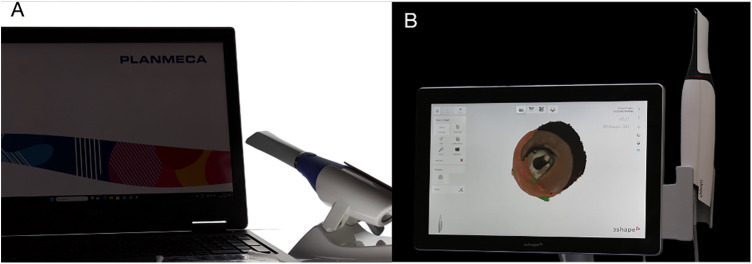
Scanners used in this study. **(A)** Planmeca Romexis®, **(B)** 3Shape TRIOS 4.

Two IOSs were used for this study: Planmeca Emerald intraoral scanner using Planmeca Romexis® software (Helsinki, Finland) (Figure 3A) and 3Shape TRIOS 4 Intraoral scanner (Copenhagen, Denmark) (Figure 3B). Both IOSs were calibrated in accordance with the manufacturer's instructions before scanning.

The study consisted of 9 groups of varying three post lengths and three post widths. Each tooth was scanned 4 times with each IOS (36 scans per scanner); Planmeca Emerald intraoral scanner using Planmeca Romexis® software (v6.4.5.202, Planmeca, Helsinki, Finland) and TRIOS 4 intraoral scanner (v23.1.4; 3Shape, Copenhagen, Denmark). A total of 72 scans were obtained, and they were exported as STL files to be used later in the 3D superimposition software.

The InEos X5 extraoral laboratory scanner (Dentsply Sirona, PA, USA) scanned the silicone replica. Afterward, to digitally compare between 2 different sets of the intraoral 3D scanners, a 3D point cloud processing software (Cloudcompare, Paris, France) program was used, which is able to handle 3D mesh.

Two trained operators took part in this step. Inter and intra-examiner reliability was estimated. To begin with the scans, standardized coding was used for both intraoral scanners and for both operators.

### 2.5 Silicone replica fabrication

**Figure 4:**
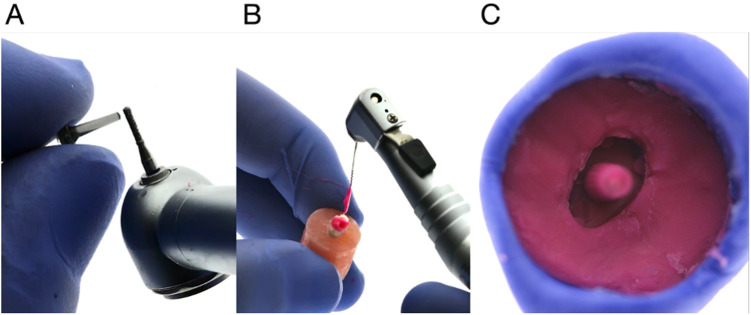
Fabrication of a silicone replica. **(A)** Plastic post roughened with straight diamond bur. **(B)** Post inserted in the tooth canal and Hydrorise putty applied in the canal using lentirulo spiral. **(C)** Putty and post assembly removed from the canal.

 After the completion of scanning, silicone replica fabrication was performed. Plastic posts (Uniclip 1 MM, DentsplySirona, Ballaigues, Switzerland) were utilized. Initially, the plastic post was shortened using a heated wax knife to create a stopper, and then it was roughened using a straight diamond bur (Figure 4A). Subsequently, with the plastic post inserted into the canal, a small amount of Hydrorise putty (Zhermack SpA, Badia Polesine, Italy) was mixed and applied in the canal using a size 25 lentirulo spiral, followed by inserting the post (Figure 4B). Then, the putty (Zhermack SpA, Badia Polesine, Italy) was applied to the head of the post.

Following this step, the putty and post assembly was removed from the canal as one piece. Upon removal, the replica was evaluated, ensuring the absence of voids, tears, and complete penetration of the impression material into the grooves (Figure 4C). The silicone replica allows for precise measurement and visualization of gaps at various interfaces, such as the buccal, palatal, mesial, and distal aspects, aiding in identifying areas of discrepancy or misfit.

The InEos X5 extraoral laboratory scanner (Dentsply Sirona, PA, USA) was used to scan the silicon replica, initially by placing the silicon replica in a magnetic circular mold and a catalyst putty was placed inside the magnetic mold to position the replica inside the mold, avoiding it from being displaced or falling from the mold during scanning. All samples were sprayed with Scan Spray (Easy Scan, Alpha Dent, Germany) before scanning to increase the quality and the accuracy of the scans by eliminating reflections and improving the appearance of the texture of the scanned replicas. All replicas were scanned and exported as STL files to be used in the 3d superimposition software.

### 2.6 Superimposition using 3D point cloud processing software

The STL files that were exported from the intraoral scanners (Planmeca and TRIOS) and the extraoral laboratory scanner (inEos X5) are then imported into the 3D point cloud processing software (Cloudcompare, EDF R&D, Paris, France). The CloudCompare software can handle both point clouds and 3D mesh files (e.g., STL, OBJ, PLY). The 3D scans were initially imported as STL mesh files, which were then converted into point clouds within CloudCompare to enable a precise, quantitative surface-to-surface comparison. This conversion facilitates the computation of cloud-to-mesh and cloud-to-cloud distances, including deviation maps and statistical parameters such as mean, standard deviation, and RMS values.

**Figure 5:**
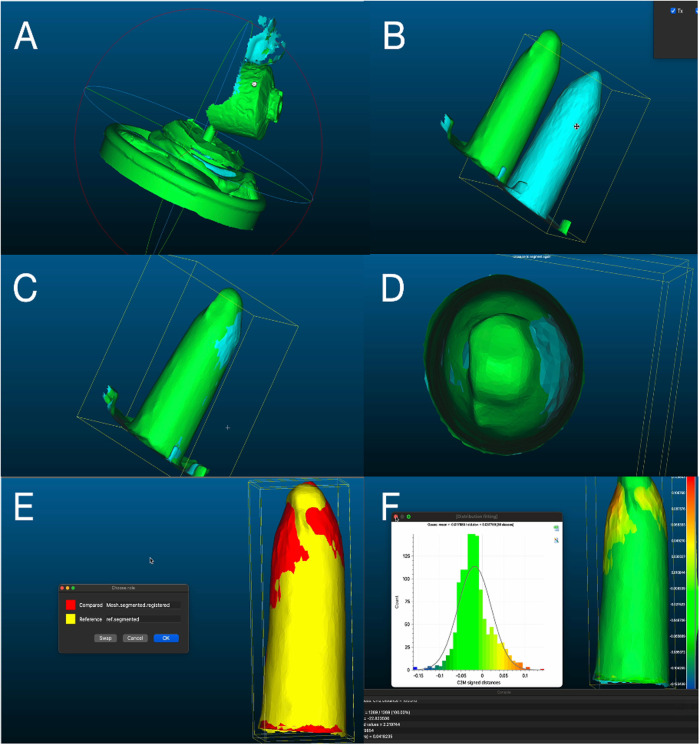
Alignment, superimposition and calculation of the discrepancy. **(A)** Each intraoral scan was imported with its matching reference (extraoral scan). **(B,C)** Using rotation/translation mode, the two scans were superimposed according to the prepared grooves. **(D)** Only the post space preparation was segmented. **(E)** Automatic alignment was used to calculate the RMS. **(F)** Compute cloud/mesh distance was used to measure the distribution fitting; three values were extracted from the software (mean, SD, and RMS).

Each scan was superimposed with its matching replica tool and adjusted manually to reduce the error (Figure 5). The superimposition was based on the entire acrylic block with the grooves, while the analysis itself focused specifically on the internal surface of the post space after segmentation. Using rotation/translation mode, the two scans were superimposed according to the prepared grooves. Only the post space preparation was segmented. The mean of the distribution fitting, standard deviation, and RMS (Root Mean Square) of the difference between the two superimposed scans were exported. Additionally, the post space length of each scanned replica and the matching scan was measured from the most cervical part to the most apical part. After the data were collected, it was documented and exported into an Excel spreadsheet for data analysis.

### 2.7 Statistical analysis

Descriptive statistics were calculated, and the normality of the data was analyzed. The statistical analyses were performed using SPSS 23.0 (SPSS Inc.). A *p*-value of <0.05 was statistically significant. Comparison of discrepancies among various post lengths and post widths with reference was done using the Kruskal–Wallis Test. Multiple comparisons of discrepancies among various post lengths and post widths were done using the Mann–Whitney *U* Test. The unit of discrepancies is mm.

The dispersion of the 3D image analysis was performed by the two examiners at two times in different sessions, 1 week apart. The two examiners had been previously trained in the use of the 3D point cloud processing software (Cloudcompare, Paris, France) program. To ensure examiner blinding, the STL files (3D images) were identified by codes and were analyzed anonymously in a random order. The intraclass correlation coefficient (ICC) was calculated for both intra-examiner and inter-examiner reliability, which considers both examiners and images as random factors.

## 3 Results

### 3.1 Descriptive results

The descriptive statistics of the RMS for each case according to the overlap in the scanning first and second examiners are shown in Table 1. The results of the Shapiro–Wilk test showed that the *p*-value was <0.05, which signified that the data were not normally distributed. In addition, the Q-Q plot, histogram, skewness, and kurtosis reflected similar results.

**Table 1:** Descriptive statistics of the root mean square (RMS) value of each case according to the overlap in the scanning of the first and second examiners.

Examiners			Statistic	Std. Error
First Examiner	Mean		0.09	0.005
	95% Confidence Interval for Mean	Lower Bound	0.08	
		Upper Bound	0.10	
	5% Trimmed Mean		0.08	
	Median		0.07	
	Variance		0.002	
	Std. Deviation		0.043	
	Skewness		1.173	0.283
	Kurtosis		1.326	0.559
Second Examiner	Mean		0.07	0.006
	95% Confidence Interval for Mean	Lower Bound	0.05	
		Upper Bound	0.08	
	5% Trimmed Mean		0.06	
	Median		0.05	
	Variance		0.003	
	Std. Deviation		0.052	
	Skewness		2.721	0.283
	Kurtosis		11.073	0.559

Furthermore, the intraclass correlation coefficient (ICC) in the main examiner (two times in different sessions, 1 week apart) and between the main examiner vs. second examiner is shown in Table 2. The main examiner's ICC was excellent; intra-examiner reliability ICC > 0.9, and inter-examiner reliability was acceptable ICC∼0.6. As the data were not normally distributed, nonparametric tests were used.

**Table 2:** Intraclass correlation coefficient (ICC) in the main examiner (two times in different sessions, 1 week apart) and between the main examiner vs second examiner.

Summary	Intraclass Correlation	95% Confidence Interval		
		Lower Bound	Upper Bound	Value
Average Measures	0.971^a^	0.924	0.989	38.030
Average Measures	0.565^b^	0.297	0.730	2.492

a Strong intraclass correlation between the two measures in the main examiner.

b Acceptable intraclass correlation between the two examiners.

### 3.2 Impact of the post length

The rank distribution of various groups of various post lengths is shown in Table 3. Kruskal–Wallis Test showed that the discrepancy between the STL scanning files (Planmeca and Trios) and the STL generated from the replica (reference) was a significant difference (*p*-value = 0.002) and the longer the post, the higher the discrepancy (Table 4).

**Table 3:** Rank distribution of various groups of various post lengths.

Ranks	Post Length	Mean Rank
Groups	6	29.88
	8	30.92
	10	48.71

**Table 4:** Comparison of discrepancies among various post lengths with reference using the Kruskal–Wallis test.

Test Statistics^a^^,^^b^	Groups
Kruskal–Wallis H	12.280
Df	2
*P*-value	0.002*

a Kruskal Wallis Test.

b Grouping Variable: Post Length.

* Statistically significant difference at *p*-value of 0.05.

Moreover, the Mann–Whitney Test revealed that there was a significant difference between the 6 mm vs. 10 mm (*p*-value = 0.001) and 8 mm vs. 10 mm (*p*-value = 0.006) lengths. However, there was no statistically significant difference between 6 mm and 8 mm (*p*-value = 0.967) lengths (Table 5).

**Table 5:** Multiple comparisons of discrepancies (mm) among various post lengths using the Mann–Whitney U test.

Post lengths	6 mm vs. 8 mm	6 mm vs. 10 mm	8 mm vs. 10 mm
Mann–Whitney U	286.000	127.000	156.000
Wilcoxon W	586.000	427.000	456.000
Z	−0.041	−3.320	−2.722
*p*-value	0.967	0.001*	0.006*

* Statistically significant difference at *p*-value of 0.05.

### 3.3 Impact of the post width

The rank distribution of various groups of various post widths is shown in Table 6. Kruskal–Wallis Test revealed that the discrepancy between the STL scanning files (Planmeca and Trios 4) and the STL generated from the replica (reference) was a significant difference (*p*-value = 0.001), and the wider the post, the smaller the discrepancy (Table 7).

**Table 6:** Rank distribution of various groups of various post widths.

Ranks	Post widths	Mean Rank
Groups	1.8	47.71
	2.1	36.96
	2.3	24.83

**Table 7:** Comparison of discrepancies among various post widths with reference using the Kruskal-–Wallis test.

Test Statistics^a^^,^^b^	Groups
Kruskal–Wallis H	14.353
Df	2
*p*-value	0.001*

a Kruskal Wallis Test.

b Grouping Variable: Post Length.

* Statistically significant difference at *p*-value of 0.05.

Moreover, the Mann–Whitney Test revealed that there was a significant difference between the 1.8 mm vs. 2.3 mm (*p*-value < 0.001) and 2.1 mm vs. 2.3 mm (*p*-value = 0.048) widths. However, there was no statistically significant difference between 1.8 mm vs. 2.1 mm (*p*-value = 0.080) (Table 8).

**Table 8:** Multiple comparisons of discrepancies (mm) among various post widths using the Mann–Whitney U test.

Post widths	1.8 mm vs. 2.1 mm	1.8 mm vs. 2.3 mm	2.1 mm vs. 2.3 mm
Mann–Whitney U	203.000	104.000	192.000
Wilcoxon W	503.000	404.000	492.000
Z	−1.753	−3.794	−1.979
*p*-value	0.080	<0.0001*	0.048

* Statistically significant difference at *p*-value of 0.05.

The unit of discrepancies is mm.

## 4 Discussion

This study investigated the accuracy of intraoral scanners at different post space preparation depths and widths for CAD/CAM fabricated post and core fabrication. The differences in deviations were precisely quantified when using the IOS technique as compared to the polyvinyl siloxane silicone replica, the traditional impression technique in post and core fabrication. The conventional dental impressions were considered the control group. In this research, we investigated the possibilities of using IOS technology to produce posts that are anatomically suitable (the least amount of preparation). These posts are recognized for several advantageous characteristics, including the preservation of a greater amount of the natural root dentin (33, 34), reducing the layer of the cement (35), minor air bubbles formation during the process (36), improved post retention (37, 38), and an increase in fracture resistance (39, 40). The results showed that the data are not normally distributed, as confirmed by the Shapiro–Wilk test. The ICC indicated excellent intra-examiner reliability (ICC > 0.9), indicating that the scanners stay consistent when used by the same operator, which is critical for clinical applications. However, the inter-examiner reliability was acceptable (ICC∼0.6), which reveals that results may vary when different operators operate the scanners. This emphasizes the significance of good training and standardization to minimize variability among operators. Statistically significant differences were observed in scanning accuracy between the group using 2.3 mm post width preparation and the other groups using 1.8 mm and 2.1 mm post width preparation, indicating variations in accuracy based on post width. However, there was no significant difference between the groups using 1.8 mm and 2.1 mm post widths. The observed discrepancies based on post length and width indicate that these factors can influence the accuracy of intraoral scanners. Longer posts may pose challenges for accurate scanning, possibly due to difficulty capturing the entire length. Conversely, wider posts may provide better surface area for scanning, leading to more accurate results. In addition, short and wide posts are generally not the most effective way to reduce the risk of vertical root fractures, as they can cause excessive dentin removal and increase stress concentration. Instead, conservative preparation is preferred, utilizing longer, narrower posts; ideally, fiber-reinforced composite (FRC) with an elasticity similar to dentin, to achieve better stress distribution (41).

In this study, 3Shape TRIOS 4 and Planmeca Emerald S intraoral scanners because they are widely used in clinical and research settings, representing two different scanning technologies and acquisition strategies. The 3Shape TRIOS employs a confocal imaging principle, while the Planmeca Emerald S uses a short-wavelength laser with active triangulation. This allowed us to compare scanners with distinct optical systems and data-processing algorithms, providing a broader and more meaningful evaluation of accuracy in post-space digitization.

The trueness (accuracy) of the TRIOS 4 scanner is approximately 20.8 ± 6.2 µm or 34.0 [14.8] µm, while its precision is around 19.75 µm (IQR 17.90–21.95 µm) (42). Another study (43) found that Planmeca Emerald had significantly lower trueness values than either the 3Shape A/S TRIOS 3 (*p*-value = 0.001). Still, the trueness (accuracy) and precision of Trios 4 and Planmeca Emerald are comparable. In this study, there was no significant difference between the Planmeca and Trios scanning systems. Clinicians should consider these factors when selecting posts for CAD/CAM direct technique procedures to ensure optimal accuracy. It is important to comprehend the elements that impact the accuracy and precision of scanners for making clinical decisions and planning treatments. Clinicians need to understand the possible limitations of intraoral scanning devices and consider variables like post widths and lengths when evaluating the appropriateness of a scanner for clinical cases. It was found that the newer scanners (e.g., TRIOS 4, Primescan) show greater resilience to distance/speed variations and enable flexible clinical workflows (44).

The incorporation of CAD-CAM technology enhances the restorative procedure, providing a safer and more effective approach, saves time, and reduces the risk of complications that may occur while preparing the root canal (45–47). The application of the CAD/CAM approach can lead to improvements in the effectiveness, efficiency, and accuracy of workflows and accuracy in prosthesis therapy. It empowers clinicians to create post and core restorations using tooth-colored materials that offer enhanced physical properties and superior mechanical qualities. This enables achieving optimal results in the restoration of endodontically treated teeth (26).

Nulty (42) conducted a study that compared the complete arch trueness of nine different intraoral scanners and four lab light digital scanners, and he reported that Ineos X5 had the best overall trueness value of (0.0 ± 1.9). Consequently, compared to all intra-oral scanners, the Ineos X5 demonstrated statistically superior precision. Researchers frequently assess the accuracy of their findings by measuring the trueness and precision, which has been a common approach in multiple studies (48, 49).

Similarly, the ferrule can be placed 3 mm above the CEJ to simulate clinical settings, resulting in larger access, potentially making it less complicated to scan the post space, especially for deeper depths (50). In 2019, the CEREC Company stated that it was feasible to scan deep gaps ≤20 mm using Primescan (Primescan, Dentsply Sirona), enabling the scan of long post spaces and the manufacture of custom posts using CAD/CAM technique. The longest post space length prepared was 20 mm, which is more likely to be found in canine teeth and long roots. They reported median RMS (Root Mean Square) values increased from 10 mm (357.1 μm) to 20 mm (897.5 μm) (50). The acceptable RMS range is 250–500 µm, and it is consistent with the clinically acceptable cement thickness for post-and-core restorations (51). However, this range represents a broader tolerance. The specific parameters of post space length and diameter influence accuracy, with longer and narrower post spaces generally having higher (less accurate) RMS values. Furthermore, longer posts (e.g., 10 mm) make the procedure more technique-sensitive and increase the potential for biomechanical failures (52, 53). And sometimes, a longer post can affect the fit and long-term clinical performance of the final restoration.

Furthermore, all IOSs use optical systems and possess some limitations. This is because light must reach every aspect of the post space preparation to ensure accurate scanning. Therefore, the quality of the digital impression of the post is influenced by the hardware and software parameters of the IOS, as the depth of focus and optimal ambient light conditions vary among different systems (54). Due to the study's *in vitro* setting, which cannot be able to accurately reflect clinical settings, this study has limitations. The efficiency of the IOS may be affected by clinical factors related to patient cooperation, illumination, saliva, oral cavity temperature, gingival mucosa and fluids, blood, limited mouth opening and accessibility, considering the precision of the fabrication of posts and cores, and the presence of adjacent teeth. Moreover, the research concentrated on a particular type of IOS, meaning its findings might not translate to other scanner varieties. Additional studies are necessary to assess how IOSs can be clinically useful for post- and core procedures across various scanner models and real-life clinical scenarios.

## 5 Conclusions

Within the limits of this study, the following conclusion can be reported:

- Increasing the length of the post preparation reduced the accuracy of the scanning, regardless of the scanner used.
- When it comes to post width, increasing the diameter of the post tends to improve the scanning accuracy.

## Data Availability

The raw data supporting the conclusions of this article will be made available by the authors, without undue reservation.
